# Head and dependent marking and dependency length in possessive noun phrases: a typological study of morphological and syntactic complexity

**DOI:** 10.1515/lingvan-2021-0074

**Published:** 2022-10-03

**Authors:** Kaius Sinnemäki, Viljami Haakana

**Affiliations:** University of Helsinki, Helsinki, Finland

**Keywords:** corpus linguistics, dependency length, dependent marking, head marking, language typology, morphological complexity, possessive noun phrase, syntactic complexity

## Abstract

The interaction of morphosyntactic features has been of great interest in research on linguistic complexity. In this paper we approach such interactions in possessive noun phrases. First, we study the interaction of head marking and dependent marking in this domain with typological feature data and with multilingual corpus data. The data suggest that there is a clear inverse relationship between head and dependent marking in possessive noun phrases in terms of complexity. The result points to evidence on complexity trade-offs and to productive integration of typological and corpus-based approaches. Second, we explore whether zero versus overt morphological marking as a measure of morphological complexity affects dependency length as a measure of syntactic complexity. Data from multilingual corpora suggest that there is no cross-linguistic trend between these measures in possessive noun phrases.

## 1 Introduction

Over the past two decades there has been an increase of interest in linguistic complexity and its distribution across languages. Of particular interest have been so-called complexity trade-offs, according to which complexity in one area of grammar (for example, morphology) would be balanced out in another area (for example, syntax). Evidence for trade-offs has been quite limited (for example, Shosted 2006) and to some extent dependent on the measure used (for example, Sinnemäki 2014a).

We measure linguistic complexity in one grammatical domain, namely, in possessive noun phrases. Semantically these constructions usually express ownership, such as *my bike*, part-whole relationships, such as *my head*, and kinship relationships, such as *my mother*. The possessee in these constructions (for example, *bike*, *head*, *mother*) is the syntactic head and the possessor (for example, *my*), if explicitly expressed, is syntactically a dependent. In this domain we are interested in the interaction of morphosyntactic features from two perspectives: i) the length of dependency within possessive noun phrases and ii) distribution of morphological marking within those constructions in terms of head versus dependent marking. Length of dependency refers to the number of intervening words between the head and the dependent in a construction and as such is a measure of syntactic complexity (for example, Gibson 1998; Yadav et al. 2020).

Head and dependent marking, or locus of marking, refers to the position of morphological marking of syntactic relations in a construction and it provides a way of approaching morphological complexity and its distribution within constructions (Nichols 1992). Four logical loci exist for morphological marking: it occurs either on the head (1a), on the dependent (1b), on both (1c), or on neither (1d). These types are called head marking, dependent marking, double marking, and zero marking, respectively. In rare instances the morphological marking is not clearly attached to either the head or the dependent; this type, detached marking (for example, Nichols 1992: 55–56), is excluded here.

(1)

a.

**Table:** 

Head marking; Indonesian (Austronesian)	
*ibu-nya*	*Suparjo*
mother-3sg.poss	Suparjo
‘Suparjo’s mother’	
(Sneddon 1996: 146)

b.

**Table:** 

Dependent marking; Swedish (Indo-European)	
*hans*	*mor*
3sg.poss	mother
‘his mother’	
(own knowledge)

c.

**Table:** 

Double marking; Finnish (Uralic)	
*hänen*	*talo-nsa*
3sg.gen	house-3poss
‘his house’	
(own knowledge)

d.

**Table:** 

Zero marking; Indonesian (Austronesian)	
*rumah*	*Tomo*
house	Tomo
‘Tomo’s house’	
(Sneddon 1996: 144)

Since the pioneering work of Nichols (1986, 1992 the locus of marking has become an important approach in language typology for classifying languages in terms of their morphological properties. This approach also enables researching complexity trade-offs in different syntactic domains by zooming in on the presence versus absence of morphological marking on the head or the dependent. Earlier research has provided incipient evidence that morphological marking of “subject” and “object” (understood as the more agent-like and the more patient-like arguments) is not distributed randomly but rather inversely either on the head (head marking/agreement) or the dependent (dependent marking/case marking) (for example, Siewierska and Bakker 1996). However, while there seems to be some trade-off between case/dependent marking and agreement/head marking in terms of “subject” and “object,” double marking is very frequent as well, to the extent that case marking is more frequent in languages that also use agreement compared to those that have no agreement (Siewierska and Bakker 2008: 299–300). In terms of a particular grammatical function there seems to be some evidence even for positive correlation: in a sample of 316 languages up to 66% of languages with case marking of the subject also have agreement. There is thus no clear unanimous evidence for an inverse relationship between case/dependent marking and agreement/head marking (see also Sinnemäki 2008: 81–84). Although these issues have been discussed in the syntactic domain of the clause, to our knowledge earlier research has not addressed the relationship between head and dependent marking in possessive constructions.

Cross-linguistic research also suggests that languages tend to minimize dependency length between the head and the dependent of a construction (for example, Gibson et al. 2019; Hawkins 2004; Jing et al. forthcoming; Liu et al. 2017). However, there has been very little research on whether case/dependent marking or agreement/head marking would interact with dependency length despite repeated calls for doing so (for example, Jing et al. forthcoming; Yadav et al. 2020). Most of the existing research has focused on a single language and on the syntactic domain of the clause and not on the phrase (for example, Ros et al. 2015; Sinnemäki and Haakana 2021), Yadav et al. (2020) providing perhaps the only initial cross-linguistic study so far.

Our aim in this paper is two-fold. We first research whether there is an inverse relationship between head and dependent marking in possessive constructions in terms of complexity. Moreover, we research whether typological analysis and multilingual corpus analysis provide converging evidence on the issue, as has been the case for some other linguistic variables (for example, Gerdes et al. 2019; Östling 2015). Typological analyses are typically based on data from descriptive grammars, while multilingual corpus analyses are based on annotated language corpora from multiple languages. If the results provided converging evidence, they would support integrating these description-based and corpus-based approaches in language typology (for example, Levshina 2019). If the results provided diverging evidence, this would call for further research on why the different sources of evidence would pull to different directions. The data for the typological analyses come from the *AUTOTYP* database (Bickel et al. 2022) and for the multilingual corpus analyses we sampled a subset from the Universal Dependencies treebanks (Nivre et al. 2018).

Our second aim is to study if zero versus overt morphological marking as a measure of morphological complexity varies in predictable ways with dependency length as a measure of syntactic complexity. For this study our data comes from a subset of the Universal Dependencies treebanks (Nivre et al. 2018). According to earlier research on sentence processing, increased distance between the head and the dependent may lead to difficulties in sentence processing. Increased dependency distance leads to greater short term memory costs and to greater costs when integrating new words into existing structure (for example, Futrell et al. 2015; Gibson 1998; Gildea and Temperley 2010). On this basis, we may assume that marking a dependency morphologically early on may facilitate identifying that dependency and assist in determining initial sentence structure (thus reducing short term memory load) or it may assist in integrating new words to existing structure at the integration site (reducing integration costs; cf. Gibson 1998; see also Ros et al. 2015; Yadav et al. 2020). If so, we could hypothesize that the presence of morphological information may also enable greater dependency distances in possessive noun phrases compared to the absence of such information. To the extent the results would support this hypothesis, they would provide evidence for a positive correlation between morphological and syntactic complexity in this construction.

The rest of the paper is structured as follows. Section 2 presents the case study on typological approach to head and dependent of marking. Section 3 presents the case studies on the multilingual corpus-based approach, first concerning the relationship between head and dependent marking and then the relationship between morphological marking and dependency length. The results are discussed and summarized in Section 4.

## 2 Case study 1: head and dependent marking in possessive noun phrases – a typological approach

### 2.1 Data and methods

The data for the typological approach to head and dependent marking in possessive noun phrases come from the *AUTOTYP* database (Bickel et al. 2022). Morphological marking in this database includes all marking whether done via bound morphemes, clitics, adpositions or isolating formatives, or morphophonological or tonal alternations (Bickel and Nichols 2007). Some languages use more than one morphological type within the same construction, such as Indonesian, which uses head marking (1a) as well as zero marking (1d) in possessive noun phrases. This kind of language-internal variation is coded in the database, so we take it into account to address both cross-linguistic and language-internal variation at the same time. The database contains 449 datapoints, that is, constructions with a particular pattern of morphological marking, from 316 different languages and averaging to 1.42 datapoints per language.

**Figure 1:**
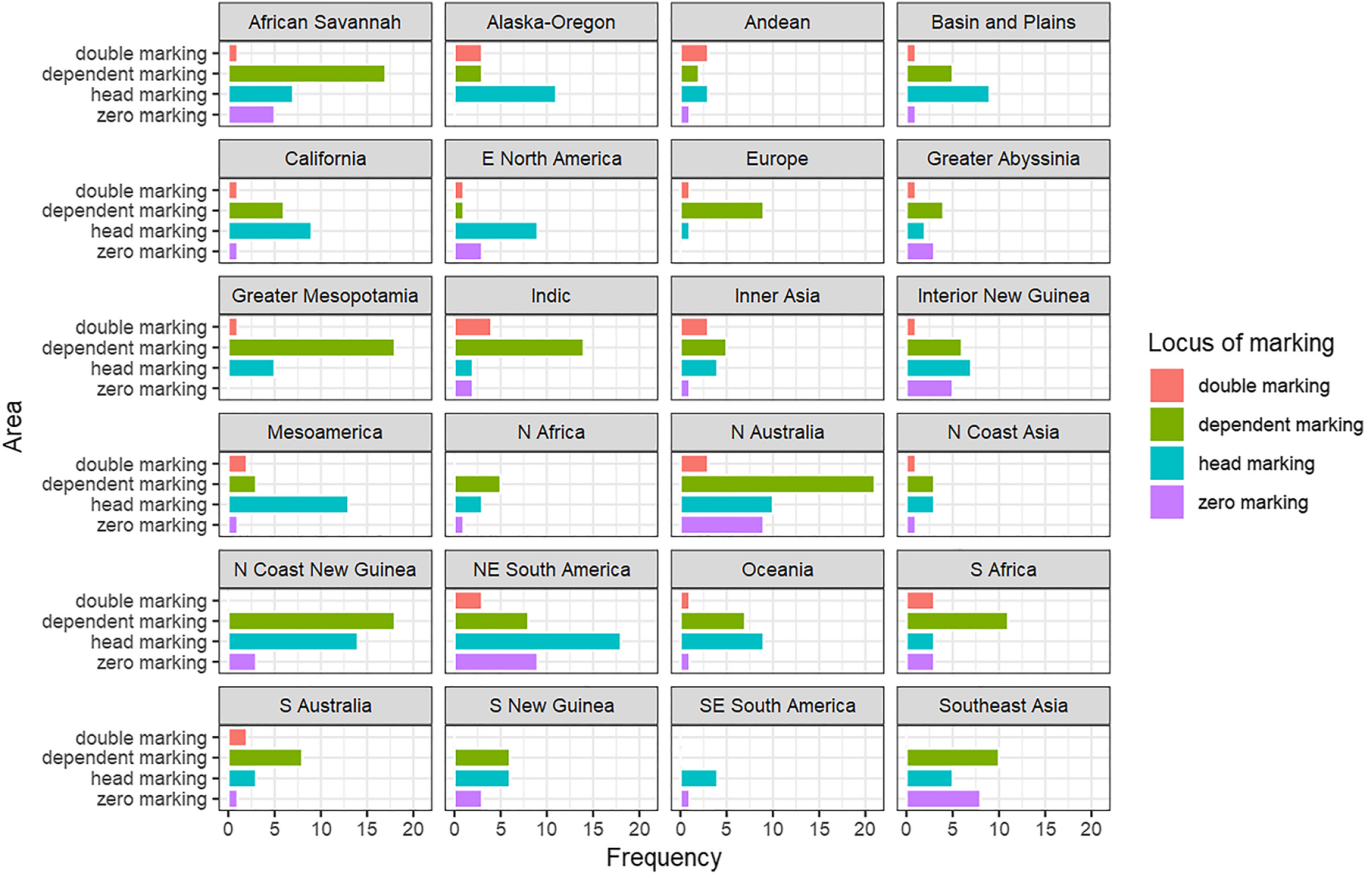
Distribution of locus of marking in possessive noun phrases over the 24 areas in the *AUTOTYP* (Bickel et al. 2022).

The datapoints are distributed in the following way: there were 63 constructions with zero marking (14%), 190 with only dependent marking (42%), 160 with only head marking (36%), and 36 with double marking (8%). This frequency distribution already points to a complementary relationship between head and dependent marking because the shares of head-marking constructions and dependent-marking constructions are much higher than those of zero marking and double marking. However, some of these datapoints come from the same language and many of the sample languages are either closely related or they have long histories of language contact with one another. It is standard practice in the field to properly address such potential confounding factors to draw more reliable conclusions (for example, Bromham et al. 2018; Siewierska 1996; Sinnemäki 2008; Winter and Grice 2021). The areal distribution of the four loci of marking is presented in Figure 1. To find out whether the relationship between head and dependent marking is independent of confounding areal and genealogical factors, we modeled their relationship with generalized linear mixed effects modeling (for applications to typological data, see Bentz and Winter 2013; Jaeger et al. 2011).

Our research question in this case study is whether there is an inverse cross-linguistic relationship between head and dependent marking in terms of complexity. The null hypothesis is that no relationship exists between them. In this typological approach we measured linguistic complexity in terms of presence versus absence of morphological marking, called “complexity as overt coding” in Sinnemäki (2014a). Based on earlier research even this kind of rough approach may give a starting point for approaching linguistic complexity in a certain grammatical domain.

In our model, head marking was selected as a binary response variable and dependent marking as a binary predictor. See the online Supplementary Materials S1 and S2 for details of the model specification and for information about preprocessing and modeling the data. The models were fitted using maximum penalized likelihood with weakly informative priors and posterior modes for estimation (Chung et al. 2013). This approach has been shown to approximate posterior median well. This method is computationally efficient because no posterior means (or medians) are used as in fully Bayesian modeling and thus no simulations are needed. *p*-Values were drawn with a likelihood ratio test and validated via parametric bootstrap (Halekoh and Højsgaard 2014). All computations and plots were produced in the *R* programming environment (R Core Team 2022); see Supplementary Material S1 for details of the packages used.

### 2.2 Results

Dependent marking had a significant negative effect on head marking (coefficient = −2.94 ± 0.35; *χ* ^2^ (1) = 41.7; *p* < 0.001). The odds ratio for dependent marking was 0.053. This means that languages that have head marking in possessive noun phrases had 94.7% lower odds of having dependent marking than those that have no head marking. The parametric bootstrap derived *p*-value was 0.0007 (using 2,000 simulations), which validates the statistical significance of the result.

To evaluate the model’s goodness-of-fit, we compared the difference in the nested model’s Akaike Information Criterion (AIC). Adding dependent marking to the null model lowered AIC by 40: this large reduction in AIC (>10) provides evidence for the model’s goodness (Burnham and Anderson 2002: 70–71). As for explanatory power, the model’s conditional *R* ^2^ was 0.43 and its marginal *R* ^2^ 0.31 (Nakagawa and Schielzeth 2013). The bulk of the model’s explanatory power thus rests on dependent marking and not, for instance, on genealogical affiliation and geographic distribution.

**Figure 2:**
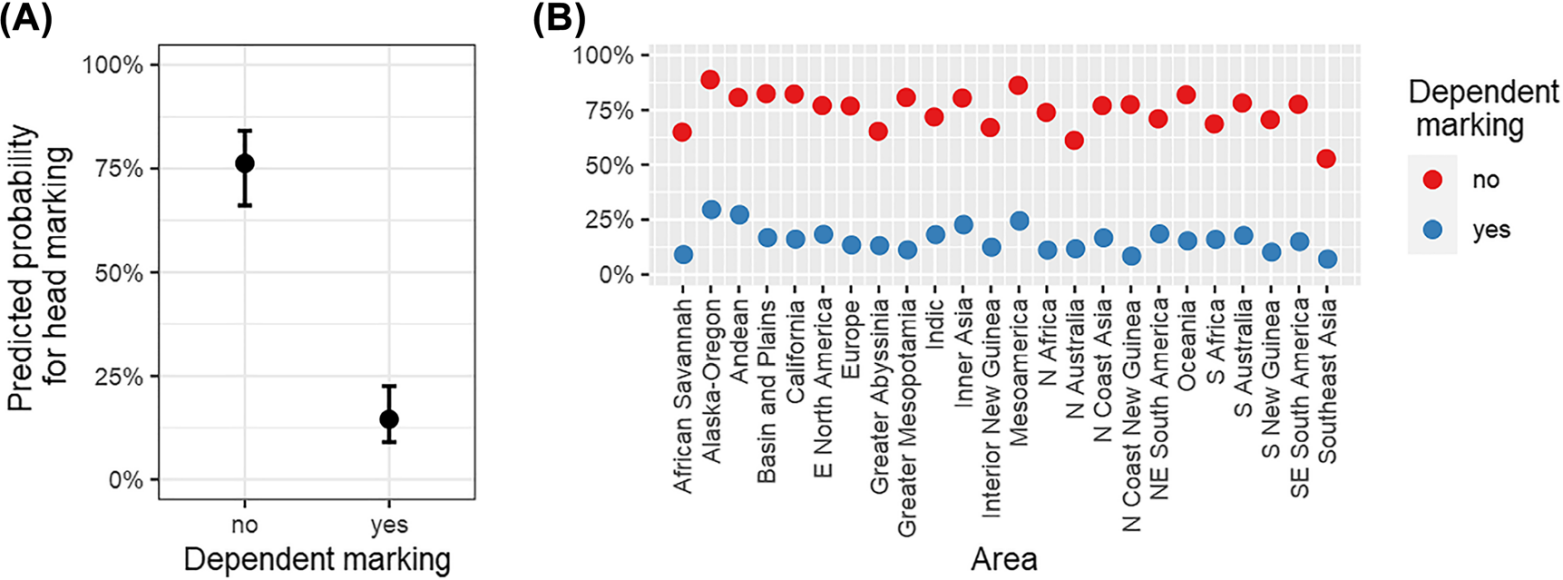
Marginal effects for the effect of dependent marking on head marking in plot A and for the random slopes for dependent marking over area in plot B.

Figure 2 presents the marginal effect plots for the model. In plot A the predictor’s values are presented on the *x*-axis and the predicted probabilities of the response (head marking) on the *y*-axis. The plot suggests a clear inverse relationship between head and dependent marking: the predicted probability of head marking is roughly 15% when the dependency relation in the possessive construction is morphologically marked on the dependent, but as high as 75% when there is no dependent marking. In plot B the predicted probabilities for head marking (*y*-axis) are given for the 24 geographical areas of the *AUTOTYP* (Bickel et al. 2022). In all 24 areas except for Alaska-Oregon and Andean, the predicted probability of head marking is 25% or less when the dependency relation in the possessive construction is morphologically marked on the dependent. In addition, in all areas the predicted probability of head marking is 50% or more when the dependency relation in the possessive construction is not morphologically marked on the dependent.1We double-checked the results by modeling language as a datapoint. The results were very similar to those reported here; see Supplementary Material S1 for more information.


Overall, these results provide clear evidence for an inverse relationship between head and dependent marking both within and across languages even after addressing the confounding genealogical and geographical factors.

## 3 Case study 2: morphological marking and dependency length in possessive noun phrases – a multilingual corpus-based approach

### 3.1 Data and methods

The data for the multilingual corpus-based approach come from a subset of 63 treebanks in 44 languages in the Universal Dependencies corpus (Nivre et al. 2018). The corpus was preselected for a shared task by the organizers of the Interactive Workshop Measuring Language Complexity held in Freiburg in September 2019. The data were analyzed by Sinnemäki and Haakana (2020) to identify possessive noun phrases, to classify them in terms of head and dependent marking, and to compute dependency length. Morphological marking was analyzed similarly to that in the *AUTOTYP* database (Bickel et al. 2022; see also Section 2). The online Supplementary Material S1 describes the preprocessing of the data in some more detail; see also Sinnemäki and Haakana (2020) for detailed analyses.

In this corpus-based approach we measured complexity in two ways, depending on the purpose. First, we provided a multilingual corpus-based counterpart for case study 1 where we correlated the presence of head marking with the presence of dependent marking. In this corpus-based analysis we measured complexity as the degree of head marking versus the degree of dependent marking and evaluated their correlation. Degree of head marking was calculated as the proportion of possessive noun phrases in a language that mark the dependency relation morphologically on the head. Degree of dependent marking was then calculated as the proportion of possessive noun phrases in a language that mark the dependency relation morphologically on the dependent. The rationale for this approach was to evaluate what is the relative importance of marking the syntactic relation in a language either on the head or the dependent. The degree of head/dependent marking is thus roughly related to the idea of functional load, which measures the relative importance of contrasts between linguistic units (see also Sinnemäki 2008). Our second purpose in this case study was to compare overt marking with dependency length. For that purpose, we measured morphological complexity in terms of “complexity as overt coding,” as in Section 2.1. This means that we simply contrasted overt morphological marking (whether via head marking, dependent marking, or double marking) with zero morphological marking (zero marking).

**Figure 3:**
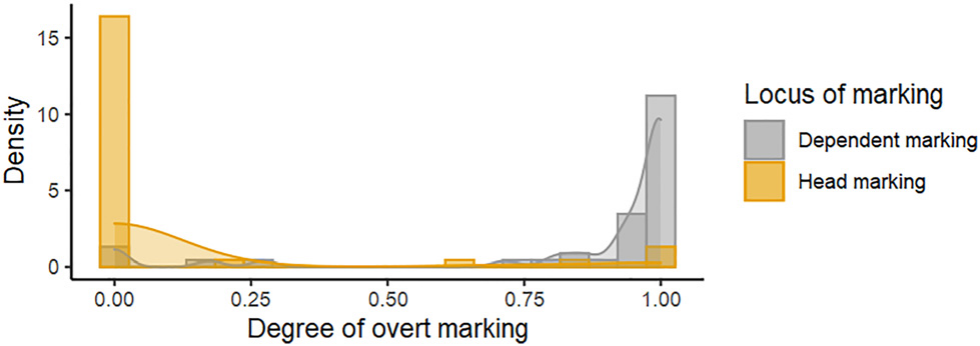
Frequency histogram and the superimposed density estimates for the degree of head marking and dependent marking.

The histogram distribution for the degree/proportion of head and dependent marking is provided in Figure 3. The proportions are not normally distributed: the bulk of the instances for head marking have small proportions, mostly close to zero and with long right tail as in gamma distribution. The bulk of the instances for dependent marking, on the contrary, have rather large proportions, mostly close to one. This discrepancy in the proportions of head and dependent marking stems from a geographical bias in the sample to the languages of Europe (cf. Sinnemäki and Haakana 2020), which are mostly dependent marking (Nichols 1992). Given these distributions, we used generalized zero-inflated gamma linear mixed effects regression (Brooks et al. 2017) to model the relationship between the degree of head marking (response) and dependent marking (predictor). P-values were estimated with a likelihood ratio test. See Supplementary Materials S1 and S2 for details of the model specification.

Dependency length was measured as the distance between the syntactic head and its dependent in a construction as a function of the number of intervening words. In the Universal Dependencies annotation each word receives a unique word index, as in Table 1 for English. Clitics may also be treated as separate words, as in the English Web Treebank, but this is to some extent specific to each treebank.

**Table 1:** A possessive noun phrase in the English Web Treebank (EWT; sent_id = answers-20111024210339AAIqPa3_ans-0002; excluding some columns; Silveira et al. 2014).

*N*	Wordform	Lemma	UPOS	Features	Head	Dependency
1	My	My	PRON	Number = Sing|Person = 1|	2	nmod:poss
				Poss = Yes|PronType = Prs		
2	boyfriend	boyfriend	NOUN	Number = Sing	4	nmod:poss
3	’s	’s	PART	_	2	case
4	birthday	birthday	NOUN	Number = Sing	6	nsubj

This means that when a dependent is morphologically marked with an item that receives a unique ID, it potentially increases dependency length. For instance, the possessive clitic *’s* in English receives its own word index and therefore increases dependency length between the possessor (*boyfriend*, index = 2) and the possessee (*birthday*, index = 4). In the UD annotation these items are identified as direct descendants of the dependent and usually annotated with the dependency relation “case”. The question then is whether dependent marking items, such as the possessive clitic *’s* in English, should be included in the count for dependency length. To address this issue, we contrasted two measures for dependency length, one that includes direct descendants of the dependent with a dependency relation “case” in the count for dependency length (DL_incl_) and one that excludes them (DL_excl_). The former measure is used in models m.incl and the latter in models m.excl.

Since our aim is to analyze the interaction of dependency length and morphological marking, we classified each dependency in possessive noun phrases in terms of morphological marking. For this reason, we did not count the average dependency length of possessive noun phrases per sentence and rather computed average dependency length in possessive noun phrases separately for zero morphological marking (zero marking) versus overt morphological marking (head, dependent, and/or double marking) in the spirit of Yadav et al. (2020: 10–11).

We modeled the relationship between dependency length and morphological marking with generalized linear mixed effects regression. Because word structures may be analyzed differently even in closely related languages, for instance, clitics may be part of the word or independent words, measures for dependency length are not necessarily commensurable across languages. For this reason, we modeled each language separately (see Futrell et al. 2020; Yadav et al. 2020; but see Jing et al. forthcoming). Dependency length was modeled as the response and morphological marking as the predictor. As was already mentioned, most sample languages have dependent marking. Five use head marking (Hungarian, Indonesian, Persian, Turkish, Uyghur) and four use double marking (Finnish, Hungarian, Turkish, and Uyghur). Moreover, the frequency of head marking and double marking is low in most of these languages. For this reason, we coded morphological marking as a binary predictor contrasting the presence versus absence of morphological marking with values “no” (zero marking) and “yes” (head, dependent, and/or double marking). In addition, we focused only on those sample languages with at least some language-internal variation in zero marking versus overt marking (see Supplementary Material S1 for details): the final sample contained 16 languages. Since dependency length is count data, we used Poisson regression to model its distribution (following Yadav et al. 2020). Sentence length was further used as a random intercept. See Supplementary Materials S1 and S2 for details of the model specification.

### 3.2 Results

**Figure 4:**
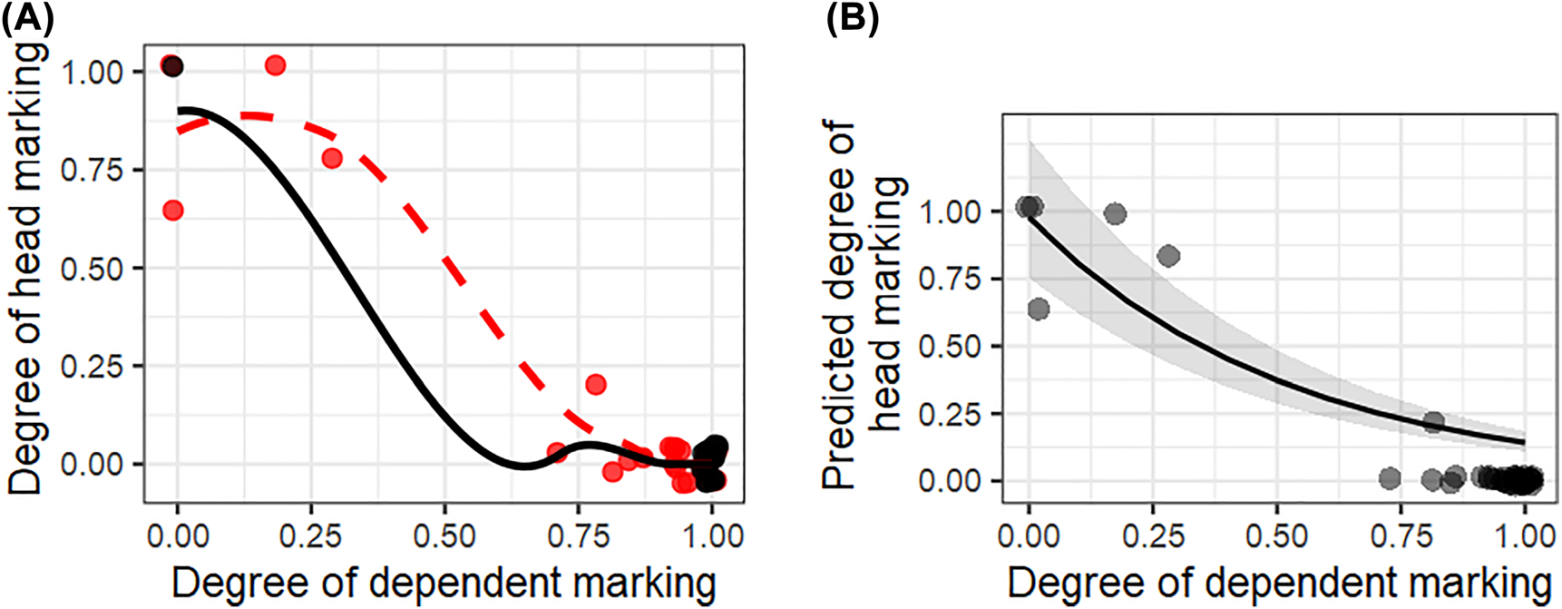
A scatter plot of the degree of dependent marking and head marking in the corpus-based approach in plot A. The black dots represent languages with no internal variation in head and dependent marking and the red dots those with some variation. The smoothed red dotted line depicts the latter languages, while the solid black line depicts the relationship between head and dependent marking in all sample languages. Plot B presents the marginal effect for the degree of dependent marking on the degree of head marking. Small jitter was added to the datapoints in both plots.

We first modeled the relationship between head and dependent marking. A scatterplot of the degrees of head and dependent marking is shown in plot A of Figure 4. The black dots represent languages in which there is no language-internal variation: all possessive noun phrases are morphologically marked on the head, as in Persian, or all of them are marked on the dependent, as in English. The red dots represent languages in which there is some language-internal variation in head and dependent marking so that the degree of head and/or dependent marking departs from 1.0, as in Indonesian. While we do not expect languages with internal variation in the degree of head and dependent marking to behave differently from those with no internal variation, separating them from one another enables us to inspect their behavior visually. The relationship between the degree of head and dependent marking in the former languages (*n* = 25) is depicted by the smoothed red dotted line in plot A, while the solid black line represents their relationship in all sample languages (*n* = 44). The smoothed lines suggest that while the relationship between the degree of head and dependent marking is not linear, there is a clear downward trend regardless of whether languages have internal variation in head and dependent marking or not. When reporting on the modeling below, all data was used.

According to the results, dependent marking had a significant negative effect on head marking (coefficient = −1.93 ± 0.01; *χ* ^2^ (3) = 21.32; *p* < 0.001). To evaluate the models’ goodness-of-fit, we compared the difference in the nested model’s AIC. Adding the interaction term to a model without it lowers AIC by 19.3: this large reductions in AIC (>10) provides evidence for the models’ goodness (cf. Burnham and Anderson 2002: 70–71). Marginal effect plot of the model is shown in plot B of Figure 4, which suggests a clear negative relationship between the variables. The coefficients and random effect variances of the model are provided in Supplementary Material S1.

**Figure 5:**
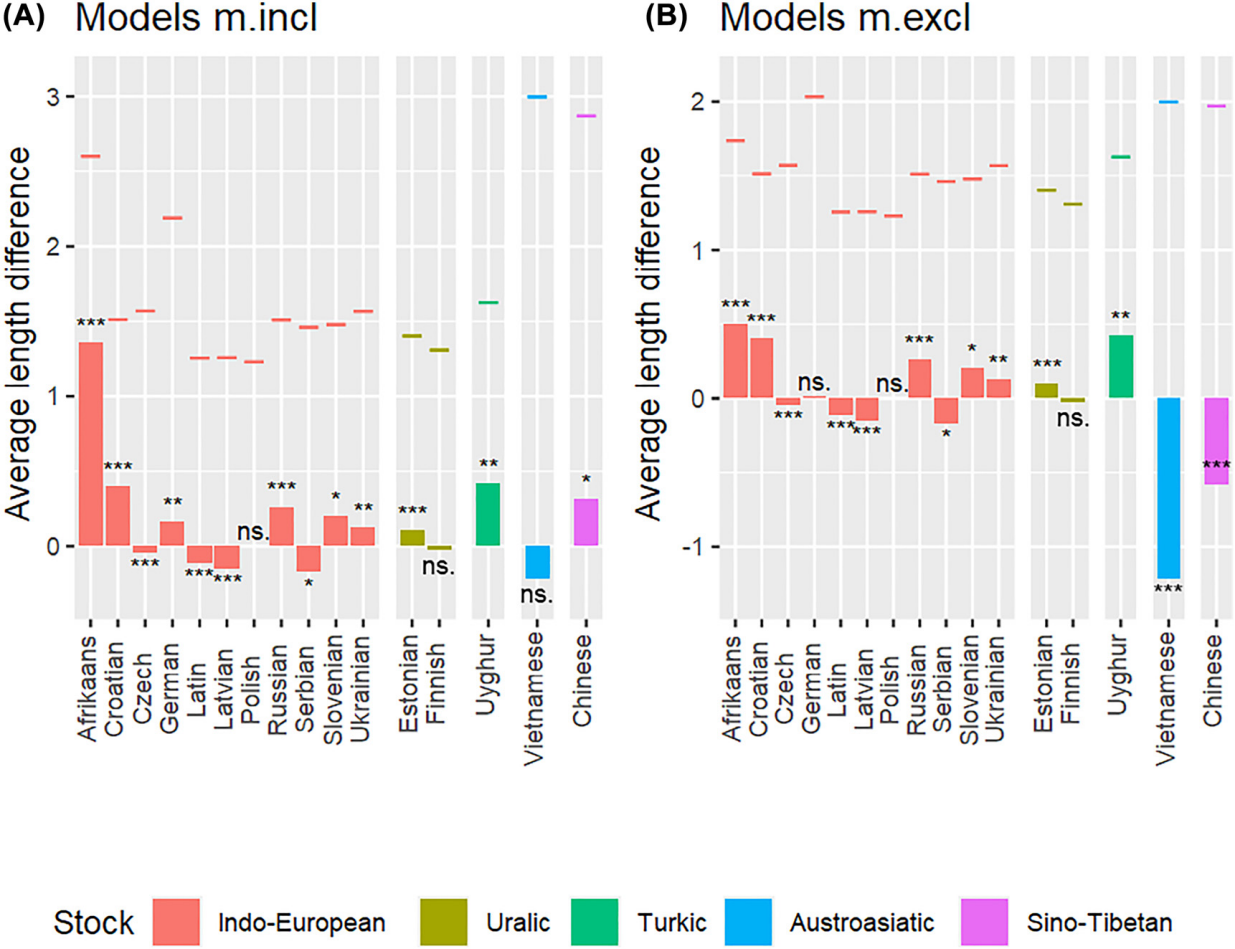
Difference in average dependency length in overt-marking and zero-marking possessive noun phrases in the sixteen sample languages; models m.incl in plot A and models m.excl in plot B (*** = *p* < 0.001; ** = *p* < 0.01; * = *p* < 0.05; ns. = not significant). The short, colored lines above the bars show the average dependency length for overt-marking constructions.

We then modeled the relationship between dependency length and morphological marking. The results are summarized in Figure 5, for models m.incl in plot A and for models m.excl in plot B. The coefficients and random effect variances of each model are provided in Supplementary Material S1. We calculated average length difference by subtracting the average dependency length in zero-marking possessive noun phrases from that in overt-marking possessive noun phrases in each language. Positive values thus indicate that the average dependency length in a language was greater for overt-marking constructions and negative values indicate that the average dependency length in a language was greater for zero-marking constructions.

The short, colored lines above the bars in Figure 5 indicate the average dependency length for overt-marking constructions. In models m.incl most of them cluster between roughly 1.2 and 1.7, but a few higher values occur between 2.2 and 3.0 in languages that use an independent word for overt marking (for example, Chinese and Vietnamese). In models m.excl, the average dependency length for overt marking varies roughly between 1.2 and 2.0. This suggests that average dependency length for overt marking constructions varies only a little across the sample languages once independent words that are used for overt marking have been excluded from the count for dependency length.

In models m.incl (plot A), overt-marked dependencies were on average longer than zero-marked dependencies in ten of the sixteen languages. This difference was significant (*p* < 0.05) in all but one language (Polish). In six languages zero-marked dependencies were on average longer than overt-marked dependencies (significant in all but Finnish and Vietnamese). There was thus no clear support for overt-marked dependencies having longer dependencies, whether including all languages (*p* = 0.45; *n* = 16; Binomial Exact Test) or just those in which the difference between overt-marked and zero-marked dependencies was significant (*p* = 0.27; *n* = 13; Binomial Exact Test). Genealogical affiliation made no difference in these distributions. There were seven Indo-European languages in which overt-marked dependencies were longer than zero-marked and four in which zero-marked dependencies were longer than overt-marked. In addition, dependency length varied strongly over morphological marking in the two sampled Uralic languages, Estonian and Finnish. Geographic region also made no clear difference in the distributions. In some languages spoken in Europe, overt-marked dependencies were longer than zero-marked, while in others it was the other way round – and likewise for languages spoken outside Europe.

In models m.excl (plot B), overt-marked dependencies were on average longer than zero-marked dependencies in nine of the sixteen languages. This difference was significant in all but two languages (German and Polish). In seven of the sixteen languages, overt-marked dependencies were on average shorter than zero-marked dependencies (significant in all but Finnish). Again, genealogical affiliation and geographic region made no difference in the distributions. Overall, there was no clear support for overt-marked dependencies having longer dependencies, whether including all languages (*p* = 0.80; *n* = 16; Binomial Exact Test) or just those in which the difference between overt-marked and zero-marked dependencies was significant (*p* = 1.00; *n* = 13; Binomial Exact Test).

All in all, the results for the two model types were similar: there was no general trend for overt-marked dependencies being longer than zero-marked. Models m.incl provided somewhat more evidence for the hypothesis but models m.excl less so. Perhaps the clearest difference between the model types was that when independent dependent-marking words were excluded (models m.excl), zero-marked dependencies became significantly longer than overt-marked in the isolating languages of Southeast Asia (Chinese and Vietnamese). Genealogical affiliation and geographic region seemed not to affect the distributions. The analyzed data thus provided no general support for the hypothesis about the relationship between dependency length (syntactic complexity) and morphological marking (morphological complexity).

## 4 Discussion

In this paper we have focused on morphological marking and dependency length in possessive noun phrases as measures of morphological and syntactic complexity, respectively. Our first aim was to research whether head and dependent marking interact in this domain. In addition, we evaluated whether morphological marking had any effect on dependency length. The results suggest three main outcomes:

1. An inverse relationship existed between the presence versus absence of head marking and dependent marking in the typological approach.
2. An inverse relationship existed between the degrees of head marking and dependent marking in the multilingual corpus-based approach.
3. Dependency length did not correlate with the presence versus absence of morphological marking systematically across languages in the multilingual corpus-based approach.

Evidence from description-based typological data (from the *AUTOTYP* database; Bickel et al. 2022) and multilingual corpus data (from the Universal Dependencies treebanks; Nivre et al. 2018) thus provide converging evidence for an inverse relationship between head and dependent marking in possessive noun phrases when the main confounding factors were addressed. The result can be taken as evidence for a complexity trade-off in the domain of possessive noun phrases: constructions in which the dependency relation between the head and the dependent is morphologically marked on the head, tend not to have dependent marking – and vice versa. The result also suggests that evidence from typological and multilingual corpus-based approaches can be productively integrated in language typology (for example, Levshina 2019), which is good news for further advancing cross-linguistic research.

As for our second aim, we tested the hypothesis that overt-marked dependencies would be on average longer than zero-marked in possessive noun phrases. This hypothesis was not borne out. Overt-marked dependencies were not consistently longer than zero-marked dependencies across the sampled languages but their correlation, where significant, seemed to depend largely on the specific language.

These results go against Yadav et al. (2020: Table S21) whose initial data suggested that non-clausal objects with case marking had longer dependencies than those without case marking (in 21 out of 22 languages; see also Ros et al. 2015; Sinnemäki and Haakana 2021). It is unclear why the data provided no evidence for our hypothesis concerning dependency length. One reason could be that Yadav et al. (2020) studied verb-object dependency, which has more flexibility in terms of word order and morphological marking compared to possessee-possessor dependency within the noun phrase. For one, word order of clausal arguments is known to be more flexible than word order of nominal modifiers (Levshina 2019). In addition, in most languages that have case marking of the object only some objects are case marked (Sinnemäki 2014b). In contrast, in 27 of the 44 sample languages at least 99% of the dependencies within possessive noun phrases were morphologically marked and in sixteen languages (the tested sample) overt marking was clearly the dominant pattern with greater than 70% incidence, even in Chinese and Vietnamese which are rather analytic and isolating in their morphology. The possessive relation thus tends to be virtually always morphologically marked in languages.

What we are seeing in possessive noun phrases may be an example of competing motivations at play in different parts of one and the same domain. The complexity trade-off between head and dependent marking may be explained in terms of economy: forms are minimized so that unnecessary overt marking – especially double marking – is avoided in languages (for example, Haspelmath 2014; Kusters 2003). But the relationship between dependency length and morphological marking appears not to be motivated by economy. The fact that almost all possessive noun phrases in our sample languages were morphologically marked may be caused by system pressure within grammars. System pressure means that grammatical rules in languages tend to target large grammatical classes rather than individual expressions (Haspelmath 2014: 197). If system pressure is so strong in possessive noun phrases across languages, it may be that there is not much room for dependency length to be affected by morphological marking, leading to largely language-specific patterns.

All in all, the results suggest evidence for an inverse relationship between head and dependent marking in terms of complexity but no evidence for a cross-linguistic correlation between dependency length (syntactic complexity) and morphological marking (morphological complexity).

## Supplementary Material

Supplementary Material

Supplementary Material
