# Supplementary material for: Head and dependent marking and dependency length in possessive noun phrases: a typological study of morphological and syntactic complexity
Source: Linguist Vanguard. 2022 Oct 3;9(Suppl1):45–57. doi: 10.1515/lingvan-2021-0074 (PMC10234274; doi:10.1515/lingvan-2021-0074)
Supplement: Supplementary file 1 — Supplementary Material [file j_lingvan-2021-0074_suppl_001.pdf]

Head and dependent marking and dependency length in possessive noun phrases: a typological study of morphological and syntactic complexity

Supplement S1: Preprocessing and modelling

Kaius Sinnemäki and Viljami Haakana
University of Helsinki

Case study 1

The AUTOTYP database version 1.0.1 (Bickel et al. 2022) provides information about head and dependent marking in different files. We used the file `LocusOfMarkingPerMicrorelation.csv`, which contains information on locus of marking and its variation both within and across languages. From this file we worked with the variable `LocusOfMarkingBinned5` and selected the following types: "D" (= dependent marking), "Ø" (= zero marking), "H" (= head marking), and "2" (= double marking). We excluded a fifth type "FloatingorClitic", which codes for floating clitics and for headward migrated dependent marking. Note that the variable `LocusOfMarkingBinned6` also contains information on locus of marking, but since the variable `LocusOfMarkingBinned5` contains more datapoints (2 048 vs. 2 040) and fewer missing data, we chose to work with it.

We modeled head marking as a binary response variable. Its values were coded in the following way: value "no" included those constructions with no form of head marking, that is, "zero marking" and "dependent marking", and value "yes" included those with any form of head marking, that is, "head marking" and "double marking". Dependent marking was modeled as a binary predictor and its values were coded in the following way: value "no" included those constructions with no form of dependent marking, that is, "zero marking" and "head marking", and value "yes" included those with any form of dependent marking, that is, "dependent marking" and "double marking". Genealogical affiliation of the sample languages and the geographical area where they are spoken were used as grouping structure to adjust the coefficients for head marking and dependent marking. Genealogical affiliation was modeled with a hierarchic structure to control for variation within and across languages: the lowest level classification in the AUTOTYP, namely languages, were embedded in the highest level of classification, namely stocks (see Bickel et al. 2022). It was not possible to model a random slope over the genealogical factors because the number of random effects would have exceeded the number of datapoints. As for geographical location of languages, we followed the AUTOTYP and classified languages into 24 areas in which they are primarily spoken (see Bickel et al. 2022) and modelled a random slope for dependent marking over these areas. These areas are illustrated in Figure S1a.

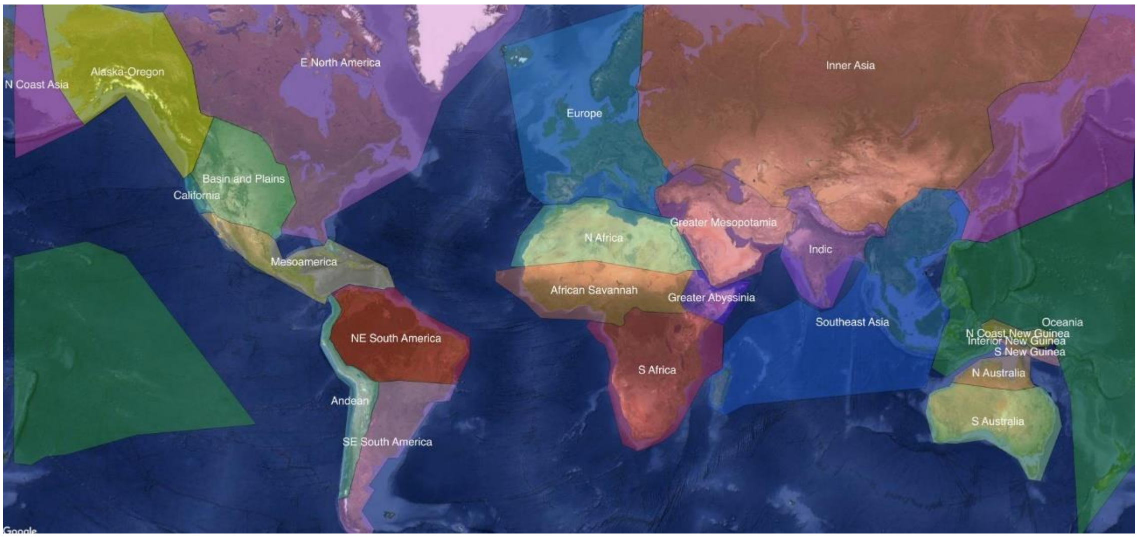

Figure S1a. The 24 areas of the AUTOTYP on a world map (Bickel et al. 2022; used under CC-BY 4.0 license).

We first ran the models using the `lme4` package (Bates et al. 2015) in R (R Core Team 2022). However, this resulted either in singular fit or models not converging. For this reason, we used generalized linear mixed effects modeling with maximum penalized likelihood by using the package `blme` in R (Chung et al. 2013). This package offers the possibility of using a prior and posterior modes for estimation yet without going into fully Bayesian inference with posterior means (or medians) from simulations; this will improve convergence and computational efficiency as well as pull the correlation terms between random intercepts and slopes away from perfect correlation (cf. Dorie 2014: 93–94). For priors we used weak (default) priors of `blme` (Chung et al. 2015). The models did not converge when using the default optimizers, and therefore we used `BOBYQA` as the optimizer in all models: this resulted in model convergence in all situations. The model specification in the `lme4` notation was as in (1):

(1)
```
head_marking ~ dependent_marking +
               (1+dependent_marking|area) +
               (1|stock/language)
```

P-values were drawn with a likelihood ratio test, and validated by a parametric bootstrap method, using 2 000 simulations (Halekoh and Højsgaard 2014). This method returns the fraction of those simulated likelihood ratio test values that are larger or equal to the observed likelihood ratio test value. The model's explanatory power was computed separately for the whole model (conditional R^2) and just for the fixed effects (marginal R^2) via the package `MuMIn` (Barton 2020). The algorithm is based on Nakagawa and Schielzeth (2013) and has been further developed by Johnson (2014), and Nakagawa, Johnson and Schielzeth (2017). All graphics in both case studies were created in R using packages COWPLOT (Wilke 2020), GGPLOT2 (Wickham 2016), SCALES (Wickham and Seidel 2020), and SJPLOT (Lüdecke 2018).

Table S1a presents the model's coefficients and Table S1b its random structure. The variances of the random terms are not too close to zero and the correlation between random intercept and random slope over area is also not close to perfect correlation.

Table S1a. Coefficients for the model in case study 1.

Parameters	Coefficient	Std. error	Z value	P value
intercept	1.167	0.256	4.561	< 0.001
dependent_marking = “yes”	–2.937	0.349	-8.406	< 0.001

Table S1b. Random effect variances for the model in case study 1.

Group	Name	Variance	Std. dev.	Correlation
language:stock	(intercept)	0.139	0.372	
stock	(intercept)	0.156	0.395	
area	(intercept)	0.552	0.743	
	dependent_marking = “yes”	0.588	0.767	-0.50

The choice of the response and the predictor variables was diachronically and thus plausibly also causally motivated: there is a diachronic trend of dependent marking developing into head marking but not the other way round (Nichols 1986). Synchronically the choice is somewhat arbitrary: either variable could have been selected as the response. Accordingly, we built a competing model by selecting dependent marking as the response and head marking as a predictor, keeping the random structure the same. The results were almost identical: head marking had a significant negative effect on dependent marking (coefficient = −2.96 ± 0.36; χ^2 (1) = 41.4; p < 0.001). Adding head marking to the null model lowered Akaike Information Criterion (AIC) by 39, which was just a little lower than the corresponding lowering of AIC in the main model (40). The model’s marginal R^2 was 0.30, which was slightly lower compared to marginal R^2 of 0.31 in the main model. What this result suggests is that there is a strong mutual relationship between head and dependent marking. Because of the diachronic relationship between the two, we focus on reporting the results for head marking as the response variable.

In these models we accounted language-internal variation in the morphological marking of possessive constructions. As discussed in Section 2 of the article, the datapoints were thus constructions with different patterns of morphological marking. To contrast a different way of counting the datapoints, we also modeled the data by focusing only on the default morphological pattern in the language. Thus, each language contributed only one datapoint. For this model we used information in the column `IsDefaultLocusOfMarking` from the file `LocusOfMarkingPerMicrorelation.csv` to select only patterns which were analyzed as default in the AUTOTYP database (with value `TRUE`). The models were otherwise built in the same way, modeling head marking as the response, dependent marking as a predictor, random intercept for stocks and random slopes for dependent marking over areas. It was not possible to model random slopes for dependent marking over stocks because the number of parameters exceeded the number of datapoints.

When modeling languages as datapoints, dependent marking had a significant negative effect on head marking (coefficient = −3.22 ± 0.61; χ^2 (1) = 22.8; p < 0.001). Adding head marking to the null model lowered Akaike Information Criterion (AIC) by 21. The model’s marginal R^2 was 0.28, which was slightly lower compared to the main model (0.31). The parametric bootstrap derived p-value was 0.0013 (using 2,000 simulations), which validates the statistical significance of the result. This result further suggests that there is a strong mutual relationship between head and dependent marking regardless of whether language-internal variation is accounted or not.

Case study 2

The data for the corpus-based analysis comes from Sinnemäki and Haakana (2020). They drafted short descriptions of how possession is expressed in the sample languages, then matched those with the Universal Dependencies annotation, and finally used the annotation to detect possessive constructions and their morphological marking in the corpus data. They delimited the analysis to constructions in which the possessed was a full noun, that is, either a common or proper noun (using the UPOS tags NOUN and PROPN, respectively). The analysis was delimited to constructions in which the possessor was personal possessive pronoun (for example, *my house*), common noun (for example, *the house of men*), or proper noun (for example, *John's house*), but demonstrative and other pronouns were excluded. The processed data contains roughly 725,000 datapoints, averaging to roughly 16,000 datapoints per language. For some languages, the data includes possessive NPs that have conjoined possessors (for example, *the house of Tim and Linda*). However, these constructions were excluded from the study on dependency length, because the conjoined possessors were not studied in all sample languages and including them could have biased the results.

For evaluating the degree of head vs. dependent marking, we used the following steps. First, we measured the presence vs. absence of the four different types of morphological marking in the identified possessive noun phrases. On top of these four types, we distinguished two subtypes of head marking, those in which the dependent was present in the construction as an independent syntactic constituent, as in (2a), and those in which it was absent, as in (2b). Constructions without a realized possessee, such as the predicate possessive pronouns *mine* and *yours*, were excluded from the study.

(2) Head marking; Acoma (Keresan)

a. *s'adyúm'ə gâam'a*
1SG.brother 3SG.house
'my brother's house'

b. *gâam'a*
3SG.house
'his house'
(Miller 1965: 177)

Second, we then counted the frequency of each morphological type, labelled here as $N_{subtype}$, where subtype is one of the following: dep = dependent marking; head = head marking; double = double marking; zero = zero marking; head0 = head marking with no dependent as a separate syntactic constituent. Third, degree (Deg) of dependent marking was calculated as the proportion of those constructions in which there was any type of dependent marking, as in (3):

(3) $$Deg(dep) = \frac{N_{dep}+N_{double}}{N_{dep}+N_{double}+N_{head}+N_{head0}+N_{zero}}$$

Degree of head marking was calculated as the proportion of those constructions in which there was any type of head marking, as in (4):

(4) $Deg(head) = \frac{N_{head}+N_{double}+N_{head0}}{N_{dep}+N_{double}+N_{head}+N_{head0}+N_{zero}}$

We measured dependency length as the distance between the syntactic head and its dependent in a construction in terms of the number of intervening words. In the CoNLL-U format of Universal Dependencies treebanks each word in a sentence receives a unique running word index, or ID. For calculating dependency length, we subtracted the running word indices for head and dependent from each other in each construction for each sentence as in Liu, Hudson and Feng (2009), see (5):

(5) $\frac{1}{n}\sum_{i=1}^{n}\left|ID_{head} - ID_{dep}\right|_i$

For modelling the relationship between head and dependent marking, we used generalized zero-inflated gamma linear mixed effects regression in R with the package `glmmTMB` (Brooks et al. 2017). This package enables modeling zero-inflated gamma distributions. For the link function, we used log link, following Bolker et al.'s (2021) recommendation.

As for the model's fixed terms, head marking was modeled as the response and dependent marking as a predictor. We used genealogical affiliation of the sample languages and the geographical area where they are spoken as grouping structure to adjust the coefficients for the intercept (the former for the conditional model, the latter for the zero-inflated model; see (6); other model specifications led to problems with convergence). Genealogical affiliation was modeled using the highest level of classification in AUTOTYP, namely stocks (see Bickel et al. 2022). As for geographical area, we followed the AUTOTYP and classified languages into 24 areas in which they are primarily spoken (see Bickel et al. 2022), as in case study 1. We tried modeling random slopes as well, but the models did not converge. For the optimizer we used `BFGS`, because it drew the random variances away from zero the most. The model specification in the `lme4` notation was as in (6):

(6)
```
head_marking ~ dependent_marking +
               (1|stock),
               ziformula = ~ 1 + (1|area)
```

P-values were drawn with a likelihood ratio test. The model's goodness-of-fit was estimated with AIC. It was not possible to compute the model's explanatory power via the package `MuMIn` (Barton 2020), because zero-inflated models are not yet implemented for that package.

Table S1c presents the coefficients for the conditional and zero-inflation models and Table S1d present the random structure of the conditional model. The variances of the random terms were not approaching zero. In the conditional model the degree of dependent marking had a significant negative effect on the degree of head marking.

Table S1c. Coefficients of the conditional and the zero-inflation models.

Parameters	Coefficient	Std error	*t*-value	*p*-value
conditional model				
intercept	-0.023	0.133	-0.17	0.862
dependent marking = yes	-1.927	0.010	-198.67	< 0.001
zero-inflation model				
intercept	1.762	0.666	2.645	0.008

Table S1d. Random effect variances for the conditional and the zero-inflation models.

Groups	Name	Variance	Std Dev
conditional model			
stock	(intercept)	0.071	0.267
zero-inflation model			
area	(intercept)	0.978	0.989

For modelling the relationship between dependency length and morphological marking, we used generalized linear mixed effects regression for each language separately, following Yadav et al. (2020). As for the model's fixed terms, dependency length was modeled as the response and morphological marking as a binary predictor with values "no", referring to zero marking (reference level), and "yes", referring to dependent marking, head marking, and double marking. We focused only on those sample languages with at least some language-internal variation in zero vs. overt marking. In some languages there was only a handful of possessive noun phrases with zero marking, while in most sample languages almost all possessive noun phrases were dependent-marked. We selected languages in which the share of possessive noun phrases with zero marking was 1 % or greater and the number of possessive noun phrases with zero marking was 30 or more. This resulted in a sample of 16 languages.

Since dependency length is count data, we modelled its distribution with Poisson regression (following Yadav et al. 2020). For modelling we used generalized linear mixed effects regression with maximum penalized likelihood by using the package `blme` in R (Chung et al. 2013), as in case study 1. With this package all the models converged, the variances were pulled away from zero, the computation was faster, and the *p*-values were typically slightly more conservative than when using the package `lme4` (Bates et al. 2015) or `glmmTMB` (Brooks et al. 2017). The models were checked for overdispersion and corrected where needed by including an observation-level random intercept. Sentence length was further used as a random intercept. The model specification with the `lme4` notation was as in (7):

(7)
```
dependency_length ~ morph_marking
                    + (1|sentence_length)
                    + (1|obs) # where needed
```

Table S1e below shows the results for model m.incl and Table S1f for model m.excl. For each language the tables provide information about the following: the average dependency length (dependency length for zero-marked dependencies subtracted from overt-marked dependencies), the modelled coefficient, the standard error, the *z*-value, the *p*-value (drawn with a likelihood ratio test), the variance of the random intercept, and the standard deviation of the random intercept.

Table S1e. Average dependency length and coefficients for the model m.incl.

Stock	Language	Avg DL	Coefficient	Std Error	*z*-value	*p*-value	Variance	Std dev
Indo-Eur.	Afrikaans	1.36	0.74	0.05	15.63	1.5e-66	0.0006	0.025
Indo-Eur.	Croatian	0.41	0.31	0.06	5.47	8.8e-9	0.0001	0.013
Indo-Eur.	Czech	-0.04	-0.02	0.01	-3.49	0.0005	0.007	0.085
Indo-Eur.	German	0.16	0.08	0.03	2.68	0.007	0.003	0.056
Indo-Eur.	Latin	-0.11	-0.09	0.03	-3.35	0.0009	0.0001	0.011
Indo-Eur.	Polish	0.001	0.002	0.05	0.04	0.98	0.0007	0.026
Indo-Eur.	Serbian	-0.17	-0.11	0.05	-2.27	0.026	0.0004	0.019
Indo-Eur.	Slovenian	0.21	0.15	0.07	2.05	0.036	0.0004	0.020
Indo-Eur.	Latvian	-0.15	-0.12	0.03	-3.38	0.0009	0.002	0.040
Indo-Eur.	Russian	0.26	0.20	0.01	13.17	6.7e-42	0.005	0.070
Indo-Eur.	Ukrainian	0.13	0.09	0.03	2.93	0.003	0.004	0.067
Turkic	Uyghur	0.42	0.29	0.11	2.64	0.006	0.056	0.238
Uralic	Estonian	0.10	0.07	0.01	6.24	3.5e-10	0.008	0.087
Uralic	Finnish	-0.03	-0.03	0.06	-0.58	0.57	0.017	0.131
Austroas.	Vietnamese	-0.21	-0.07	0.10	-0.66	0.50	0.003	0.052
Sino-Tib.	Chinese	0.32	0.12	0.05	2.52	0.010	0.002	0.047

Table S1f. Average dependency length and coefficients for the model m.excl.

Stock	Language	Avg DL	Coefficient	Std Error	*z*-value	*p*-value	Variance	Std dev
Indo-Eur.	Afrikaans	0.50	0.34	0.05	6.97	4.3e-13	0.001	0.029
Indo-Eur.	Croatian	0.41	0.31	0.06	5.47	8.8e-9	0.0002	0.013
Indo-Eur.	Czech	-0.04	-0.02	0.01	-3.49	0.0005	0.007	0.085
Indo-Eur.	German	0.01	0.01	0.03	0.17	0.86	0.002	0.047
Indo-Eur.	Latin	-0.11	-0.09	0.03	-3.35	0.0009	0.0001	0.011
Indo-Eur.	Polish	0.001	0.002	0.05	0.04	0.98	0.0007	0.026
Indo-Eur.	Serbian	-0.17	-0.11	0.05	-2.27	0.026	0.0004	0.019
Indo-Eur.	Slovenian	0.21	0.15	0.07	2.05	0.036	0.0004	0.020
Indo-Eur.	Latvian	-0.15	-0.12	0.03	-3.38	0.0009	0.002	0.040
Indo-Eur.	Russian	0.26	0.20	0.01	13.17	1.3e-42	0.005	0.070
Indo-Eur.	Ukrainian	0.13	0.09	0.03	2.93	0.003	0.004	0.067
Turkic	Uyghur	0.42	0.29	0.11	2.64	0.006	0.056	0.238
Uralic	Estonian	0.10	0.07	0.01	6.24	3.5e-10	0.008	0.087
Uralic	Finnish	-0.03	-0.03	0.06	-0.58	0.57	0.017	0.131
Austroas.	Vietnamese	-1.21	-0.47	0.10	-4.50	1.7e-5	0.006	0.078
Sino-Tib.	Chinese	-0.58	-0.26	0.05	-4.94	1.3e-6	0.004	0.062

References

Barton, Kamil. 2020. MuMIn: Multi-Model Inference. R package version 1.43.17. https://CRAN.R-project.org/package=MuMIn.

Bates, Douglas, Martin Maechler, Ben Bolker & Steve Walker. 2015. Fitting linear mixed-effects models using lme4. *Journal of Statistical Software* 67(1). 1–48. https://doi.org/10.18637/jss.v067.i01.

Bickel, Balthasar, Johanna Nichols, Taras Zakharko, Alena Witzlack-Makarevich, Kristine Hildebrandt, Michael Rießler, Lennart Bierkandt, Fernando Zúñiga & John B. Lowe. 2022. The AUTOTYP database (v1.0.1). https://github.com/autotyp/autotyp-data/tree/0.1.2.

Bolker, Ben et al. 2021. GLMM FAQ. Available at https://bbolker.github.io/mixedmodels-misc/glmmFAQ.html (accessed 12 May 2021).

Brooks, Mollie E., Kasper Kristensen, Koen J. van Benthem, Arni Magnusson, Casper W. Berg, Anders Nielsen, Hans J. Skaug, Martin Maechler & Benjamin M. Bolker. 2017. glmmTMB balances speed and flexibility among packages for zero-inflated generalized linear mixed modeling. *The R Journal* 9(2). 378–400.

Chung Yeojin, Sophia Rabe-Hesketh, Vincent Dorie, Andrew Gelman & Jingchen Liu. 2013. A nondegenerate penalized likelihood estimator for variance parameters in multilevel models. *Psychometrika* 78(4). 685–709. https://doi.org/10.1007/s11336-013-9328-2.

Chung, Yeojin, Andrew Gelman, Sophia Rabe-Hesketh, Jingchen Liu & Vincent Dorie. 2015. Weakly informative prior for point estimation of covariance matrices in hierarchical models. *Journal of Educational and Behavioral Statistics* 40(2). 136–157. https://doi.org/10.3102/1076998615570945.

Dorie, Vincent. 2014. *Mixed Methods for Mixed Models: Bayesian Point Estimation and Classical Uncertainty Measures in Multilevel Models*. New York, NY: Columbia University dissertation.

Halekoh, Ulrich & Søren Højsgaard. 2014. A Kenward-Roger approximation and parametric bootstrap methods for tests in linear mixed models: The R package pbkrtest. *Journal of Statistical Software* 58(10). 1–30. https://doi.org/10.18637/jss.v059.i09.

Johnson, Paul C. D. 2014. Extension of Nakagawa & Schielzeth's R^2_{GLMM} to random slopes models. *Methods in Ecology and Evolution* 5. 944–946. https://doi.org/10.1111/2041-210X.12225.

Liu, Haitao, Richard Hudson & Zhiwei Feng. 2009. Using a Chinese treebank to measure dependency distance. *Corpus Linguistics and Linguistic Theory* 5(2). 161–174. https://doi.org/10.1515/CLLT.2009.07.

Lüdecke, Daniel. 2018. sjPlot: Data visualization for statistics in social science. R package version 2.6.2. https://CRAN.R-project.org/package=sjPlot and https://zenodo.org/record/2400856#.YvEBaHZBxaQ.

Miller, Wick R. 1965. *Acoma Grammar and Texts*. Berkeley: University of California Press.

Nakagawa, Shinichi & Holger Schielzeth. 2013. A general and simple method for obtaining R^2 from Generalized Linear Mixed-effects Models. *Methods in Ecology and Evolution* 4(2). 133–142. https://doi.org/10.1111/j.2041-210x.2012.00261.x.

Nakagawa, Shinichi, Paul C. D. Johnson & Holger Schielzeth. 2017. The coefficient of determination R^2 and intra-class correlation coefficient from generalized linear mixed-effects models revisited and expanded. *Journal of the Royal Society Interface* 14. 134. https://doi.org/10.1098/rsif.2017.0213.

Nichols, Johanna. 1986. Head-marking and dependent-marking grammar. *Language* 62(1). 56–119. https://doi.org/10.2307/415601.

R Core Team. 2022. *R: A language and environment for statistical computing*. R foundation for statistical computing, Vienna, Austria. https://www.R-project.org.

Sinnemäki, Kaius & Viljami Haakana. 2020. Variation in Universal Dependencies annotation: A token-based typological case study on adpossessive constructions. In Marie-Catherine de Marneffe, Miryam de Lhoneux, Joakim Nivre & Sebastian Schuster (eds.), *Fourth Workshop on Universal Dependencies (UDW 2020)*, 158–167. Barcelona (online): The Association for Computational Linguistics. https://www.aclweb.org/anthology/2020.udw-1.0.

Wickham, Hadley. 2016. *ggplot2: Elegant Graphics for Data Analysis*. New York, NY: Springer-Verlag.

Wickham, Hadley & Dana Seidel. 2022. scales: Scale Functions for Visualization. R package version 1.2.0. https://CRAN.R-project.org/package=scales.

Wilke, Claus O. 2020. cowplot: Streamlined plot theme and plot annotations for 'ggplot2'. R package version 1.1.1. https://CRAN.R-project.org/package=cowplot.

Yadav, Himanshu, Ashwini Vaidya, Vishakha Shukla & Samar Husain. 2020. Word order typology interacts with linguistic complexity: A cross-linguistic corpus study. *Cognitive Science* 44(4). e12822. https://doi.org/10.1111/cogs.12822.
